# Adherence to 2020 ESC recommendations on physical activity in a population with different cardiovascular risk levels: A prospective population-based study from the CoLaus/PsyCoLaus study

**DOI:** 10.1016/j.pmedr.2024.102743

**Published:** 2024-04-24

**Authors:** Rafaël Hauser, Roxane de la Harpe, Peter Vollenweider, Roger Hullin, Julien Vaucher, Pedro Marques-Vidal, Marie Méan

**Affiliations:** a Department of Medicine, Division of Internal Medicine, Lausanne University Hospital and University of Lausanne, Rue du Bugnon 46, 1011 Lausanne, Switzerland; b Division of Cardiology, Lausanne University Hospital and University of Lausanne, Rue du Bugnon 46, 1011 Lausanne, Switzerland

## Abstract

**Introduction:**

In 2020, the European Society of Cardiology (ESC) recommends 150 min of moderate or 75 min of vigorous-intensity PA per week. While general population PA adherence is suboptimal, its status among those with previous ASCVD or high ASCVD risk remains unknown. We aimed to assess objective adherence to ESC PA recommendations using accelerometer-based measurement among these populations.

**Methodology:**

We used data from the Swiss CoLaus|PsyCoLaus cohort study (2014–2016). PA was measured using a 14-day wrist accelerometer. Adherence was defined as > 80 % of recommended PA achievement. Adherence was investigated separately among participants with previous ASCVD and among cardiovascular risk groups (based on the Systematic Coronary Risk Evaluation SCORE-1 and more recent SCORE2) with simple and multivariable logistic regressions. Participants’ characteristics were also evaluated as independent factors after adjustment.

**Results:**

We studied 1867 participants (median age: 61.2 years, 51.3 % female). ESC PA Adherence reached 55.5 % overall, and 37 % in those with previous ASCVD. Multivariable analysis showed no significant association between previous ASCVD or high cardiovascular risk and PA adherence (Odds ratio adjusted [OR_a_] 0.9, 95 % Confidence Interval [CI] 0.6–1.4 and OR_a_ 0.7, 95 % CI 0.4–1.2, respectively). Age (≥60 years old), obesity, smoking, chronic renal disease, hypertension, diabetes and benzodiazepine use were significantly associated with lower likelihood of PA adherence in multivariable logistic regression.

**Conclusion:**

Adherence to ESC PA guidelines, particularly in participants with higher cardiovascular risk, was poor. Since PA adherence was associated with modifiable risk factors (e.g., obesity, smoking, and benzodiazepine use), maintained efforts to implement the ESC recommendations are advised.

## 1 Introduction

Atherosclerotic cardiovascular disease (ASCVD), including acute myocardial infarction, symptomatic coronary artery, and ischemic stroke, accounts for 30 % of all global deaths. (Balakumar et al., 2016 Nov) Studies suggest that the recommended physical activity (PA) levels decrease the risk of ASCVD by about 15 %, making it a cost-effective prevention measure. (van Trier et al., 2022 Jan, Fiuza-Luces et al., 2018 Dec) Therefore, PA is the corner stone of cardiovascular disease prevention. The 2020 European Society of Cardiology (ESC) guidelines recommends 150 min of moderate or 75 min of vigorous-intensity PA per week. (Pelliccia et al., 2021 Jan 1).

Previous studies have shown suboptimal adherence to PA recommendations in general population across diverse regions (between 20–50 %). (Mf et al., 2015, Arias-Palencia et al., 2015, Bennie et al., 2019, Lee and Vitiello, 2022 Jul 28) One major limit in literature is a lack of standardized and objective PA measurement with most studies assessing self-reported PA. (Loyen et al., 2016 Jun) Previous studies showed that overall PA adherence was lower in participants with cardiovascular risk factors than without (37 % vs. 47 %) and that the overall adherence decreased with increasing number of cardiovascular risk factors. (Mf et al., 2015) However, the adherence to ESC PA guidelines in a population with previous ASCVD is unknown. It has also been shown that individual characteristics such as age, BMI, chronic renal disease, depression, education level and household income are all negative factors associated with PA adherence. (Jefferis et al., 2014 Apr, Martinez-Harvell et al., 2020 Dec).

Therefore, we aimed to assess the association between participants who experienced an ASCVD event or those at high risk of developing an ASCVD according to a clinical risk score assessment, and their objective level of adherence to 2020 ESC PA guidelines, measured using objective accelerometer-based measurement. Our hypothesis was that people with previous ASCVD or high cardiovascular risk score would have received higher therapeutic education, subsequently engaging in more lifestyle changes, including increased adherence to PA. We also investigated association between individual characteristics and PA adherence to identify potential targets for enhancing therapeutic PA education.

## 2 Methodology

### 2.1 Selection of participants

The CoLaus|PsyCoLaus study is a prospective European population-based cohort exploring the determinants of ASCVD. (Firmann et al., 2008 Mar, Verhoog et al., 2019 Nov) Briefly, a simple, non-stratified random sample of 6733 people from the population of Lausanne (Switzerland) was recruited between 2003 and 2006. The CoLaus/PsyColaus sample was on average one year younger than the base population of Lausanne, due to an under-representation of participants aged over 65 years while no differences were found for gender distribution. (Firmann et al., 2008 Mar).

**Fig. 1:**
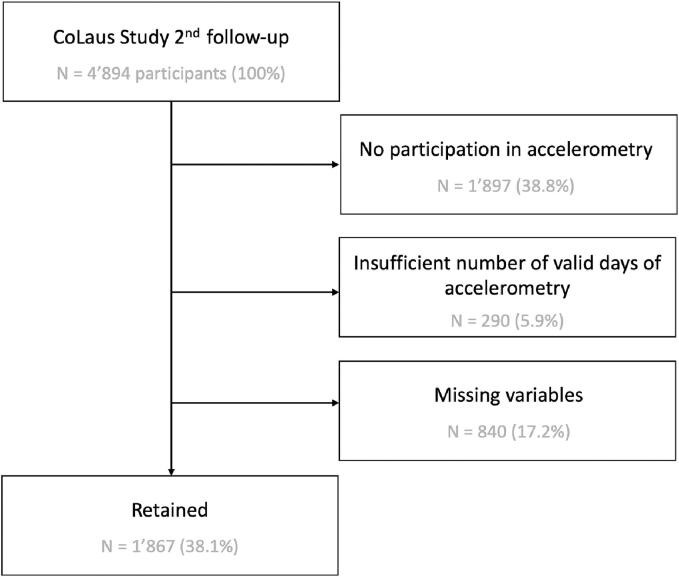
Study flow chart. CoLaus Study second follow-up: 2014–2017. 1897 participants of the CoLaus second follow-up did not participate in accelerometry. 18 participants were excluded for a non-wear of the accelerometer more than 20 % of the time. 272 participants were excluded for wearing the accelerometer less than 7 days. 840 participants were excluded for missing co-variables (10 missing BMI, 72 missing working status, 264 missing income details, 373 missing depression self-assessment, 82 missing alcohol consumption). The total retained number of participants was 1867.

All the 4894 participants present at the second follow-up (2014–2016) were included in this study. Participants were excluded if they did not participate in accelerometer module, had less than 7 consecutive days of valid accelerometery data or > 20 % of non-wear time during the measurement period, or had at least one missing baseline characteristic that was included in the current analyses. (Fig. 1). We excluded all individuals with at least one missing value relevant to the current analyses.

### 2.2 Ethical statement and consent

The local Ethics Commission approved the CoLaus|PsyCoLaus study (www.cer-vd.ch; project number PB_2018-00038, reference 239/09) and all participants provided written informed consent. The study was performed in agreement with the Helsinki Declaration and in accordance with the applicable Swiss legislation.

### 2.3 Physical activity measurement

PA was assessed by a wrist-worn triaxial accelerometer, called GENEActiv which was previously validated against reference methods and used for physical activity assessment in a large cohort study. (White et al., 2016 Dec 9, Esliger et al., 2011 Jun) The accelerometers, pre-programmed with a 50 Hz sampling frequency, were attached to the participants’ right wrist. After a short training period, participants were requested to wear the device continuously, day and night, for 14 days in their free-living conditions. A valid day was defined as ≥ 10 h (i.e., 600 min-epoch) and ≥ 8 h (i.e., 480 min-epoch) of wear-time on weekdays and weekend days, respectively, based on previous studies. (Gubelmann et al., 2017 Dec, Gubelmann et al., 2018) Accelerometry data were downloaded using the GENEActiv software version 2.9 (GENEActiv, Activinsights Ltd, United Kingdom www.activinsights.com) and collapsed into 1-min epoch intervals (min-epoch). The data was then analyzed using the R-package GGIR version 2.3–3 (http://cran.r-project.org; R-code used is described in Aebischer and al study. (Aebischer et al., 2022 Jul 1) Non-wear time was defined by the software based on built-in specific criteria. (van Hees et al., 2014 Oct, Migueles et al., 2019 Sep 1) Three different intensity cutoffs were defined previously by specific ranges of average acceleration per epoch in gravitational units (mG) for the device attached to the right wrist; (Esliger et al., 2011 Jun, 1.1 Cut-points expressed in gravitational units (this vignette) [Internet]. [cited, 2024) i. Low-intensity physical activity (LPA) (<100 mG); ii. Moderate-intensity physical activity (MPA) (100 to 399 mG) and iii. Vigorous-intensity physical activity (VPA) (≥400 mG). Previous studies have shown that measuring site does not influence measurement. (Dieu et al., 2017) Total time spent in each PA’s intensity category per day was average for all valid days. We required 7 consecutive days (5 weekdays and 2 weekend days of valid accelerometry data were required) of valid accelerometry data to accurately calculate adherence to physical activity recommendations per week. The first day was discarded to reduce bias, as participants would be tempted to increase their physical activity as they were being monitored (Hawthorne effect). This left a maximum of 13 days for measurement, and not all participants wore the accelerometer for the total amount of time. Hence, as assessment of physical activity could start any weekday, it was not possible to obtain data for two whole weeks and it was decided to use only a complete week of measurement starting the second day of measurement. Therefore, we limited our analysis to the accelerometer data collected between the second and eighth day.

### 2.4 Adherence to 2020 ESC guidelines recommendations on physical activity

The 2020 ESC guidelines on sports cardiology and exercise in patients for ASCVD prevention was used to construct adherence to PA guidelines.([) Participants were considered adherent if they achieved more than 80 % of the recommended PA per week (i.e., 150 min/week of moderate-intensity PA or 75 min/week of vigorous-intensity PA), which translates to a minimum of 120 min of moderate-intensity or 60 min of vigorous-intensity PA per week. These recommendations were consistent with those in place in 2012, corresponding to the period of recruitment of the participants. (Perk et al., 2012 Jul) As the duration of PA intensity for constructing the three physical activity categories included all accelerometer data, including bouts less than 10 min, we chose not to consider the minimum 10 min bouts criteria recommended by the 2012 guideline. This would have led to an under-estimation of quantity of PA and therefore adherence. (Eser et al., 2022) Moreover, a recent study demonstrated that any length of time of PA is sufficient to impact cardiovascular mortality. (Stamatakis et al., 2022 Dec).

### 2.5 Characteristics measurement

Participants were invited to attend the outpatient clinic at Lausanne University Hospital in the morning following an overnight fast for baseline and follow-up clinical assessment, questionnaire completion, and blood sample collection. Demographic (i.e., age and gender), socio-economic and lifestyle factors and medication usage were recorded. For this study, we used employment status, categorized as “current worker” if participants reported being currently engaged in a professional activity during the follow-up appointment and monthly income level. Monthly income levels were classified based on Swiss Federal Statistical Office standards (i.e., less than 5000 CHF for low monthly income level and more than 9500 CHF for high income). (Seuils de revenu déterminant l’appartenance au groupe à revenus moyens -, 2023) Smoking status was defined as “current smoker” versus “never or former smoker.” Current alcohol consumer was determined using the Swiss federal definition of chronic risk consumption (≥2 units per day for men and ≥ 1 unit per day for women). Depression was identified as either antidepression medication use or a CES-D depression score > 17 points for men and > 23points for women. (Fuhrer and Rouillon, 1989) Blood pressure (BP) was measured three times on the left arm after at least 10 min of rest in the seated position, with the average of the last two measurements used for analyses. Hypertension was defined according to the 2013 ESC guidelines, effective the second follow-up, as a systolic BP (SBP) ≥ 140 mm Hg and/or a diastolic BP (DBP) ≥ 90 mm Hg during the visit and/or presence of anti-hypertensive drug treatment. (Authors et al., 2013 Jul 21) Low-density lipoprotein cholesterol (LDL) levels were calculated according to Sampson’s equation. (Sampson et al., 2020 May 1) Dyslipidemia was defined as either statin use, or level of LDL or total cholesterol suggestive of familial hypercholesterolemia, respectively > 4.9, >7.9 mmol/l or severe hypertriglyceridemia > 5 mmol/l, or if participant’s LDL cholesterol exceeded the recommended threshold based on their clinical risk score using the 2016 guidelines for management of dyslipidemia, effective at the second follow-up (i.e., LDL > 4.9 for low risk and LDL > 2.5 for intermediate and high risk, and LDL > 1.8 for very high risk). (Catapano et al., 2016 Oct 14) Diabetes mellitus was defined as fasting plasma glucose ≥ 7.0 mmol/L and/or presence of oral hypoglycemic or insulin treatment. (Firmann et al., 2008 Mar).

### 2.6 Previous cardiovascular event and cardiovascular risk levels

ASCVD comprising acute myocardial infarction (AMI), symptomatic coronary artery disease with greater than 70 % stenosis treated by percutaneous coronary intervention or coronary artery bypass graft (CHD), and ischaemic stroke (including transient ischaemic attack) that happened before PA measurement were included in the analyses as previous ASCVD. ASCVD events were comprehensively collected and independently adjudicated. (Beuret et al., 2021 Apr 10).

Systematic COronary Risk Evaluation (ESC-SCORE) (Perk et al., 2012 Jul), a clinical cardiovascular risk score recommended at the time of the follow-up (2014–2016) to categorize individuals according to their risk level, was computed for each participant (Supplementary Methodology **for more details**). We used SCORE1 to be closer to the recommendations the participants might have received at the time of the follow-up (2014–2016). Nevertheless, the most recent clinic cardiovascular risk score from ESC, i.e., SCORE2, would probably better reflect the real risk of our population, since it was based on more contemporary cohorts and larger data sets (Supplementary Methodology **for more details**). (SCORE2 working group and ESC Cardiovascular risk collaboration, 2021 Jul 1) A secondary analysis with SCORE2 was conducted to confirm absence of difference in our primary results.

No cardiovascular risk factor was considered if participants presented all the following characteristics: no dyslipidaemia, no diabetes, no hypertension, no obesity (BMI ≥ 30 kg/m^2^), no current smoking, no previous ASCVD. (Grundy et al., 2019 Jun 25).

### 2.7 Statistical analysis

Statistical analyses were conducted using Stata v.16.2 (Stata Corp, College Station, TX, USA). All statistical tests were 2-sided, a p-value (*P)* < 0.05 was considered significant. We also considered significant 95 % confidence intervals that did not cross 1 for regression analyses. Participants’ characteristics were described according to their adherence on ESC PA guidelines and according to their cardiovascular risk levels. Categorical variables were summarized as number of participants with column percentages, and continuous variables as means with standard deviation (SD) for symmetric distribution and median with interquartile [IRQ] for asymmetric distribution. Pearson chi-square (for categorical variables), one-way ANOVA analysis (for multi-categorical variables), Student’s T test (for symmetric continuous variables) and Mann-Whitney test (for asymmetric continuous variables) were used to assess differences in participants’ baseline characteristics according to adherence. For multi-categorical variables, linear relation to the outcome was also tested with contrast linear hypotheses.

The proportion of adherence to ESC PA guidelines for cardiovascular prevention was computed in i. All study participants; ii. For participants with previous ASCVD; iii. For each category of SCORE; and iv. In participants without cardiovascular risk factors. Associations between previous ASCVD or cardiovascular risk levels and adherence were then assessed with bivariate logistic regression.

A multivariable logistic regression model with characteristics significantly associated with PA adherence in bivariate analyses was computed to test adjusted association between previous ASCVD or cardiovascular risk levels and adherence. We also evaluated the evidence for an association between participants' characteristics and adherence to physical activity (PA) as significant independent factors after adjustment. This analysis was conducted to identify potential targets for enhancing the therapeutic education of PA.

As we hypothesized that people with previous ASCVD should be more adherent, we assume that the closer the event was to the physical activity assessment, the more adherent the participants should be, given that secondary prevention recommendations would have been recently provided. A sensitivity bivariate and multivariable analysis was then computed to precise association between recent (i.e., less than 2 years before the PA accelerometer module) vs. old (i.e., more than 2 years before the PA accelerometer module) ASCVD event and adherence. The cutoff of 2 years was selected to ensure an adequate number of events per adherent category, enabling the proper conduct of the analyses. We also assessed a sensitivity bivariate and multivariable analysis between having no cardiovascular risk factors and adherence, as well as a sensitivity multivariable analysis stratified by gender. Finally, a sensitivity analysis considering adherence as 100 % of the recommended physical activity was also computed.

We analyzed 1867 participants from the CoLaus|PsyCoLaus cohort (Fig. 1), 114 had a previous ASCVD (6.1 %). The median age was 61.2 years [15.8] and 957 (51.3 %) were females (Table 1**, see** Supplementary Table 1 for characteristics of excluded participants,).

**Table 1:** Characteristics of participants according to adherence to the 2020 ESC physical activity guidelines for cardiovascular prevention.

	All	Adherence to ESC PA guidelines		
	(%) [IQR]	No	Yes	P-value
**N**	1867 (1 0 0)	831 (44.5)	1036 (55.5)	
**Women**	957 (51.3)	416 (43.5)	541 (56.5)	0.35
**Age** (*years)*	61.2 [15.8]	64.7 [15.6]	58.3 [12.7]	<0.001
**Current worker**	1135 (60.8)	397 (35)	738 (65)	<0.001
**Monthly household income**				
Low (up to 4′999 CHF)	434 (23.3)	247 (29.7)	187 (18.1)	<0.001
Moderate (5′000 to 9′499 CHF)	813 (43.6)	359 (43.2)	454 (43.8)	
High (more than 9′500 CHF)	620 (33.2)	225 (27.1)	395 (38.1)	
**Current smoker**	324 (17.4)	171(52.7)	153(47.3)	0.001
**BMI***(kg/m^2^)*	26.2 [5.6]	27.1 [5.7]	25.5 [5.1]	<0.001
**Current alcohol consumer**	529 (28.3)	228 (43.1)	301 (56.9)	0.44
**Arterial Hypertension**	423 (22.7)	238 (56.3)	185 (43.7)	<0.001
**Dyslipidemia**	1170 (62.7)	613 (52.4)	557 (47.6)	<0.001
**Diabetes mellitus**	165 (8.8)	114(69.1)	51(30.9)	<0.001
**GFR (ml/min/m^2^)**	81.1 ± 14.5	77.4 ± 15.6	84.1 ± 12.9	<0.001
GFR > 90	561 (30.1)	191 (34)	370 (66)	<0.001
GFR 90–60	1160 (62.1)	529 (45.6)	631 (54.4)	
GFR < 60	146 (7.8)	111 (76)	35 (24)	
**Cardiovascular treatments**				
Statin therapy	335 (17.9)	212 (63.3)	123 (36.7)	<0.001
Diabetic therapy	117 (6.3)	86 (73.5)	31 (26.5)	<0.001
Antihypertensive therapy	470 (25.2)	279 (59.4)	191 (40.6)	<0.001
**Depression**	355 (19)	179 (50.4)	176 (49.6)	0.013
**Benzodiazepine**	134 (7.2)	89 (66.4)	45 (33.6)	<0.001
**Previous ASCVD**	114 (6.1)	71 (62.3)	43 (37.7)	<0.001
<2 years before baseline	25 (21.9)	17 (68)	8 (32)	0.5
**Risk according to ESC-SCORE 1**	3.8 ± 4.8	5.4 ± 5.6	2.5 ± 3.4	<0.001
Low risk (<1%)	637 (34.1)	179 (28.1)	458 (71.9)	<0.001
Intermediate-risk (1 % − 5 %)	579 (31)	237 (40.9)	342 (59.1)	
High-risk (5 % − 10 %)	256 (13.7)	146 (57)	110 (43)	
Very high-risk (≥10 %)	395 (21.2)	269 (68.1)	126 (31.9)	

Results express the number of participants (%), mean ± SD or median [IQR]. Percentages are expressed by row. P-values were derived using Pearson chi-square or Student’s T test where appropriate.

Adherence to European Society of Cardiology (ESC) physical activity (PA) guidelines were defined as 120 min of moderate-intensity or 60 min of vigorous-intensity per week.

Current alcohol consumer was defined using Swiss federal definition of chronic risk consumption (≥ 2 units per day for men and ≥ 1 unit per day for women.

GFR was estimated according to Chronic Kidney Disease Epidemiology Collaboration equation (CKD-EPI).

ASCVD: atherosclerotic cardiovascular disease; overweight HDL: high-density lipoprotein; LDL: low-density lipoprotein; GFR: glomerular filtration rate; IQR, interquartile range; SD: standard deviation.

**Fig. 2:**
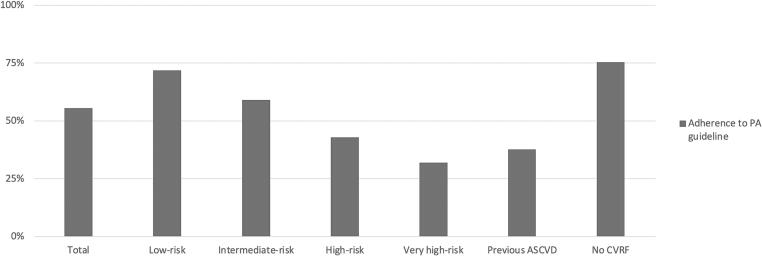
Proportion of adherence to the 2020 ESC PA guidelines, overall, according to cardiovascular risk levels and previous atherosclerotic cardiovascular disease. We used the Systematic COronary Risk Evaluation (ESC-SCORE 1) to categorize individuals according to their 10-years risk level (Low-risk < 1 %, Intermediate risk > 1 % and < 5 %, High risk > 5 % and < 10 %, Very high-risk > 10 %). No cardiovascular risk factor (CVRF) = absence of dyslipidaemia, diabetes, hypertension, obesity (BMI >= 30), current smoking, and previous ASCVD.

**Fig. 3:**
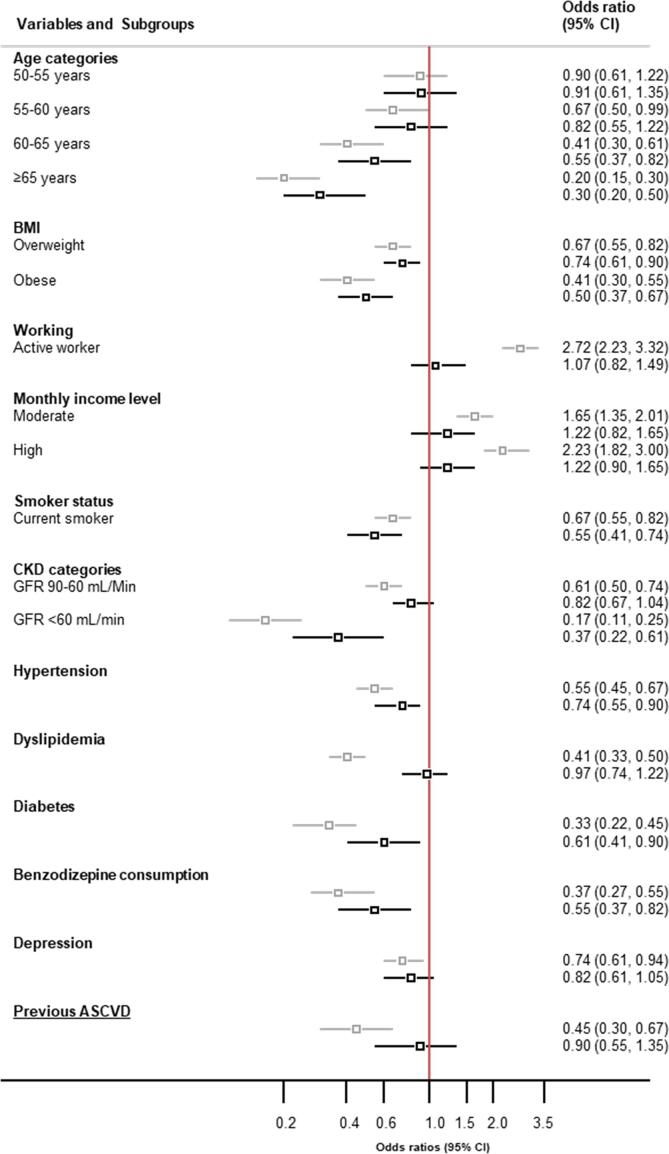
Crude and adjusted odds ratio estimates for previous atherosclerotic cardiovascular disease and individual’s characteristics with adherence to the 2020 ESC physical activity guidelines. Logistic regressions were used to test associations with adherence to ESC PA guidelines. For each characteristic, a bivariate analyze is first presented (light grey squares and lines). Second, a multivariable analysis, adjusted for age categories, BMI categories, Chronic Kidney Disease (CKD) categories, working status, income categories, smoking status, hypertension, diabetes, dyslipidemia, depression, use of benzodiazepine and previous ASCVD, has been performed (dark squares and lines). Lines which do not cross 1 correspond to significant associations. Reference category was 45–55 years for age; normal and underweight for BMI, up to 2′990 CHF for monthly household income, >90 ml/min/m2 GFR for chronic renal disease. Reference for dichotomized variable was “No”. ASCVD: atherosclerotic cardiovascular disease; GFR: glomerular filtration rate.

**Fig. 4:**
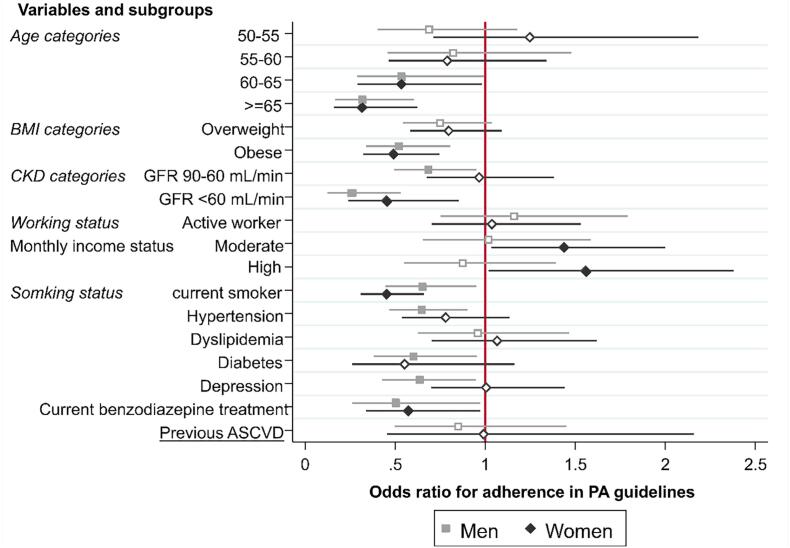
Adjusted odds ratio estimates for previous atherosclerotic cardiovascular disease and individual’s characteristics with adherence to the 2020 ESC physical activity guidelines. stratified by gender. Multivariable logistic regressions adjusted for age categories, BMI categories, Chronic Kidney Disease (CKD) categories, working status, income categories, smoking status, hypertension, diabetes, dyslipidemia, depression, use of benzodiazepine and previous ASCVD, were used to test associations with adherence to ESC PA guidelines stratified by gender (men with light grey squares and lines and women with grey diamonds and lines). Hollow squares/diamonds correspond to non-significant associations. Reference category was 45–55 years for age; normal and underweight for BMI, less than 5000.- for low monthly household income, >90 ml/min/m2 GFR for chronic renal disease. Reference for dichotomized variable was “No”. ASCVD: atherosclerotic cardiovascular disease; GFR: glomerular filtration rate.

About half (55.5 %) of our sample was adherent to PA guidelines, with 37.7 % of adherence in participants with previous ASCVD (Fig. 2). Participants with previous ASCVD adhered significantly less compared to those without ASCVD in bivariate analysis, but there was no more significant association in multivariable analysis (Odds ratio adjusted [OR_a_] 0.9, 95 % CI 0.6–1.4, p-value = 0.6) (Fig. 3), even when stratified by gender (Fig. 4). Furthermore, the likelihood of PA adherence did not significantly differ whether previous ASCVD was recent or not (OR_a_ 0.2, 95 %CI 0.1–1.0, p-value = 0.05) (Supplementary Table 2).

Proportion of adherence to PA guidelines differed significantly between the different cardiovascular risk groups (with p-value < 0.001; Fig. 2) with a significant linear tendency (i.e., lower adherence when cardiovascular risk level raises; p-value < 0.001). There was no significant association to adherence for individuals at very high cardiovascular risk category compared to those at low-risk category (Supplementary Table 2).

Table 1 shows participants’ characteristics according to adherence to PA guidelines. Mean of age, mean of BMI, proportion of current smoker, proportion of benzodiazepine, antidepressants, statin therapy, antidiabetic, anti-hypertensive consumption and proportion of depression, diabetic, dyslipidemia, hypertension and chronic renal disease were significantly lower in the adherent group. Participants with higher monthly income were more likely to be adherent, with a significantly linear tendency, as well as those currently working.

In multivariable analysis, age (≥60 years old), BMI (obese individuals), current smoker, chronic renal disease (GFR ≤ 60 ml/min/m^2^), hypertension, diabetes and benzodiazepine use were associated with a lower likelihood of adherence (Fig. 3). Age, BMI, current smoker and chronic renal disease and benzodiazepine use remained associated with a lower likelihood of adherence when stratified by gender. Hypertension and diabetes remained associated with a lower likelihood of adherence only for men. Depression, which was not associated with a lower likelihood of adherence in overall multivariable analysis, was significantly associated with a lower likelihood of adherence for men, whereas a moderate or high monthly income was significantly associated with a higher likelihood of adherence for women (Fig. 4).

The secondary analysis when assessing cardiovascular risk category according to SCORE2 showed similar results.

## 3 Discussion

According to our results, the adherence to ESC PA guidelines in a Swiss population-based is relatively low. Overall, one in two participants is adherent and only a third of people having suffered from previous ASCVD adheres to PA recommendations. The proportion of participant’s adherence to PA decreases of 2,5-fold when cardiovascular risk level raises (75 % of participants at low risk compared to 30 % of participants at very-high risk were adherent). We found no association between previous ASCVD and adherence to PA, nor with increased cardiovascular risk level when adjusting with confounders. Increasing age remains the individual characteristic associated with the lowest likelihood of adherence. Interestingly, some modifiable factors, such as high BMI, current smoking or benzodiazepine use are also associated with a lower likelihood of adherence.

Our results are in accordance with previous studies showing similar adherence, ranging from 20 to 50 % in general population (Mf et al., 2015, Arias-Palencia et al., 2015, Bennie et al., 2019, Lee and Vitiello, 2022 Jul 28, Jefferis et al., 2014 Apr, Cheng et al., 2022 Sep 16). However, a minority of studies assessed PA levels using objective measures. (Mf et al., 2015, Jefferis et al., 2014 Apr) We found that the PA adherence was lower in participants with higher cardiovascular risk, similarly to a previous study assessing PA with objective measures. (Mf et al., 2015) In this mainly male Spanish cohort of workers aged 40–55, the likelihood of adherence was the lowest when participants cumulated 4 cardiovascular risk factors (OR 0.19 95 % CI 0.11–0.32).

Contrary to our assumption and despite the alleged therapeutic education that participants having suffered from an ASCVD should have received, these participants were not more likely to adhere to PA recommendations, even if the ASCVD event was recent.

This could be explained by the fact that these participants are facing barriers to PA such as age (Jefferis et al., 2014 Apr, Moschny et al., 2011 Nov 2), overweight (Ball et al., 2000 Jun) and diabetes (Brazeau et al., 2008 Nov 1). These factors were strongly associated with a lower likelihood of adherence in our multivariable analysis. These barriers could strongly limit the impact of therapeutic education, despite the educative explanations the benefits of PA, previously described as an incentive for PA adherence. (Spiteri et al., 2019 Sep 1) We could question to which extend such explanation has been given to every participant with a previous ASCVD or high cardiovascular risk in this cohort. Indeed, recent studies highlighted the considerable benefits of cardiac rehabilitation programs in reducing mortality, morbidity, and hospital readmissions, alongside enhancing quality of life and psychological well-being. However, it is concerning that only half of eligible individuals were advised to participate. (Kotseva et al., 2018 Aug 1) Our findings may underscore a notable gap in healthcare provision regarding physical activity counselling for patients with high cardiovascular risk or diseases and in future whether it should be strengthened. It is also unclear whether low adherence to PA recommendations in patients with high cardiovascular risk or manifest cardiovascular disease is only a result of low adherence or is due to physical inability to perform the required physical activity. In this last case, counselling for more physical activity would be unlikely to improve adherence.

Individual characteristics that we found significantly associated with lower adherence were similar to previous studies. In the literature, chronic health conditions, such as chronic renal disease, hypertension, diabetes and depression all decrease independently the likelihood of PA adherence. (Jefferis et al., 2014 Apr, Vancampfort et al., 2017 Jan 18) A higher economic status has been proved to be associated with a higher level of adherence, this was only seen for women in our study. (Martinez-Harvell et al., 2020 Dec, Ramadhan et al., 2019 Nov 15) Interestingly, we found that consumption of benzodiazepine had a significant lower likelihood of adherence. This was not previously described in the literature, although an increased risk for decline in physical function have been associated with benzodiazepine use. (Gray et al., 2002 Jun) This association was also significant in sensitivity analysis stratified by sex. Benzodiazepines might be an interesting modifiable risk factor for PA adherence. We found also that current smokers had a lower likelihood of PA adherence, although the proportion of smokers did not differ according to cardiovascular risk levels or previous ASCVD in our cohort. Moreover, there is a growing body of literature examining the use of exercise interventions as an add-on therapy to smoking cessation programs. (Ussher et al., 2019, Bize et al., 2010 Dec 1) PA educational therapy targeted at smokers could improve both adherence to PA recommendations and smoking cessation, thereby majoring the risk reduction of ASCVD. (Inoue-Choi et al., 2022 Sep 1).

## 4 Study strength and limitations

To the best of our knowledge, this is one of the largest studies assessing the adherence to recent PA guidelines in a population with different cardiovascular risk level using objective measurement, i.e., accelerometers. Another strength of this study is the well described prospective cohort, derived from a population-based sample defined 10 years before the current assessments.

This study also has several limitations. First, it is important to note that the guidelines on PA have evolved through the years, although the PA guidelines have been very similar since 2012. Secondly, the GENEActiv used in the study has been shown to overestimate PA and underestimate sedentary behavior. (Rosenberger et al., 2016 Mar) Moreover, results derived from the accelerometer are strongly dependent on the cut-offs used to define LPA, MPA, VPA. (Barros et al., 2021 Sep, Vähä-Ypyä et al., 2022 Aug 26) In addition, using 80 % threshold for adherence classification instead of 100 % may underestimate poor adherence to ESC guidelines. However, this classification is unlikely to bias our results, as misclassification of participant with 80 to 99 % of adherence is unlikely to systematically differ between exposed groups (e.g., participants with previous ASCVD versus those without). Moreover, studies demonstrated that even modest amounts of physical activity, as low as one-third of guidelines, are associated with beneficial health outcomes. (Jerome et al., 2023 Jun 20, Kraus et al., 2019 Jun) Thus, the use of the 80 % threshold for adherence classification, a common practice in observational studies of medication adherence (Shah et al., 2023), may better reflect real-world scenarios. Finally, only 44.5 % of the participants seen in the second follow-up of the CoLaus/PsyCoLaus study participated in the module assessing the physical activity. Although this rate is comparable to participant rates reported in other accelerometry studies in the literature (i.e., UK Biobank 45 %, Whitehall II 42 %) (Menai et al., 2017 Apr, Watts et al., 2023 Feb 16), the retained sample might no longer be representative of the general population. The retained sample might be subject to the volunteer bias. Nevertheless, as we did not find any positive adherence, this bias is less likely to alter our results. Finally, the results are limited by the observational design of the study making reverse causation possible. (Lee et al., 2021 Mar).

## 5 Conclusion

Adherence to ESC PA guidelines was poor; only a third of participants with previous ASCVD were adherent. Being at high cardiovascular risk or having a previous ASCVD event was not associated with higher PA adherence, although these participants should have received more therapeutic education. Knowing the influence PA has on the cardiovascular risks profile of participants, such as a reducing both LDL and TG (Bajer et al., 2019 Nov 1), it is important to identify rapidly participants at risk of non-adherence, with modifiable risk factors, to guide them towards a structure specialized in helping patients achieve their PA goals. Therefore, modifiable risk factors for PA adherence such as higher BMI, smoking, and benzodiazepine use should be systematically checked to continue efforts to implement the ESC recommendations.

## 6 DATA AVAILABILITY

The data of CoLaus|PsyCoLaus study used in this article cannot be fully shared as they contain potentially sensitive personal information on participants. According to the Ethics Committee for Research of the Canton of Vaud, sharing these data would be a violation of the Swiss legislation with respect to privacy protection. However, coded individual-level data that do not allow researchers to identify participants are available upon request to researchers who meet the criteria for data sharing of the CoLaus|PsyCoLaus Datacenter (CHUV, Lausanne, Switzerland). Any researcher affiliated to a public or private research institution who complies with the CoLaus|PsyCoLaus standards can submit a research application to research.colaus@chuv.ch or research.psycolaus@chuv.ch. Proposals requiring baseline data only, will be evaluated by the baseline (local) Scientific Committee (SC) of the CoLaus and PsyCoLaus studies. Proposals requiring follow-up data will be evaluated by the follow-up (multicentric) SC of the CoLaus|PsyCoLaus cohort study. Detailed instructions for gaining access to the CoLaus|PsyCoLaus data used in this study are available at www.colaus-psycolaus.ch/professionals/how-to-collaborate/.

## Funding

The CoLaus|PsyCoLaus study was supported by research grants from GlaxoSmithKline, the Faculty of Biology and Medicine of Lausanne, the Swiss National Science Foundation (grants 3200B0–105993, 3200B0-118308, 33CSCO-122661, 33CS30-139468, 33CS30-148401, 33CS30_177535 and 3247730_204523) and the Swiss Personalized Health Network (project: Swiss Ageing Citizen Reference). The funders had no role in the design of the study; in the collection, analyses, or interpretation of data; in the writing of the manuscript; or in the decision to publish the results.

## CRediT authorship contribution statement

**Rafaël Hauser:** Writing – original draft, Visualization. **Roxane de la Harpe:** Writing – review & editing, Writing – original draft, Formal analysis. **Peter Vollenweider:** Writing – review & editing, Data curation. **Roger Hullin:** Writing – review & editing, Supervision. **Julien Vaucher:** Writing – review & editing. **Pedro Marques-Vidal:** Writing – review & editing, Validation. **Marie Méan:** Writing – review & editing, Supervision, Conceptualization.

## Declaration of competing interest

The authors declare that they have no known competing financial interests or personal relationships that could have appeared to influence the work reported in this paper.

## Data Availability

Data will be made available on request.
