# Supplementary material for: Adherence to 2020 ESC recommendations on physical activity in a population with different cardiovascular risk levels: A prospective population-based study from the CoLaus/PsyCoLaus study
Source: Prev Med Rep. 2024 Apr 24;42:102743. doi: 10.1016/j.pmedr.2024.102743 (PMC11068929; doi:10.1016/j.pmedr.2024.102743)
Supplement: Supplementary Data 1 [file mmc1.docx]

**Adherence to 2020 ESC recommendations on physical activity in a population with different cardiovascular risk levels: a prospective population-based study from the CoLaus/PsyCoLaus Study**

**Supplementary Material**

**Supplementary Methodology**

**ESC Systematic Coronary Risk Equation 1**

Concerning the ESC risk prediction model (ESC-SCORE1), the four different categories of risk were defined as low (<1%), moderate (>1% to <5%), high (>5% to <10%) and very high (>10%). Switzerland is classified in low CV risk countries and we therefore used the according chart ([ESC - SCORE charts)](https://www.escardio.org/static_file/Escardio/Subspecialty/EACPR/Documents/score-charts.pdf). Participants with i) moderate chronic kidney disease (eGFR 30-59 ml/min/1.73 m^2^) or ii) LDL-C > 7.9 mmol/l or iii) blood pressure > 180/110 mm Hg or iiii) diabetes mellitus were also attributed to the high-risk category without calculating their CV risk with SCORE. Participants were attributed to the very-high risk category if they were known for i) CVD at baseline (defined as history of acute coronary syndrome, coronary or other arterial revascularisation procedure, stroke or TIA, peripheral arteriopathy or aortic aneurism), or ii) type 1 or 2 diabetes mellitus with target organ damage or associated CV risk factors, or iii) severe chronic kidney disease (eGFR <30 ml/min/1.73m^2^).

**ESC Systematic Coronary Risk Equation 2**

SCORE2, was constructed with SCORE2 equation for individuals younger than 70 years old and SCORE2-OP equation for those equal or older than 70, as recently established for low-risk countries (i.e. Switzerland is a low-risk country)(SCORE2-OP working group and ESC Cardiovascular risk collaboration, 2021)(SCORE2 working group and ESC Cardiovascular risk collaboration, 2021)(Visseren et al., 2021)

Three different categories of risk were defined according to age, as low/moderate (<2,5% for people age <50 years; <5% for people aged ≥50 and <70; <7.5% for people aged ≥ 70), high (2,5-7,5% for people aged <50 years; 5-10% for people aged ≥50 and <70; 7.5-15% for people aged ≥ 70) and very high (≥7,5% for people aged <50 years; ≥10% for people aged ≥50 and <70; ≥15% for people aged ≥ 70). Participants were attributed to the very-high risk category if they were known for i) CVD at baseline (defined as history of acute coronary syndrome, coronary), or ii) on lipid-lowering therapy, or iii) total cholesterol >7,5mmol/L or LDL-cholesterol >4,9mmol/L, or iv) diabetes and eGFR (CKD-EPI) <60 mL/min/1.73m2 v) eGFR (CKD-EPI) < 45 mL/min/1.73m2. Participants were attributed to the high-risk category if they were at low-moderate risk category, but suffered from i) diabetes, or ii) eGFR (CKD-EPI) < 60 mL/min/1.73m2.

**References**

SCORE2 working group and ESC Cardiovascular risk collaboration. (2021). SCORE2 risk prediction algorithms: New models to estimate 10-year risk of cardiovascular disease in Europe. *European Heart Journal*, *42*(25), 2439–2454. https://doi.org/10.1093/eurheartj/ehab309

SCORE2-OP working group and ESC Cardiovascular risk collaboration. (2021). SCORE2-OP risk prediction algorithms: Estimating incident cardiovascular event risk in older persons in four geographical risk regions. *European Heart Journal*, *42*(25), 2455–2467. https://doi.org/10.1093/eurheartj/ehab312

Visseren, F. L. J., Mach, F., Smulders, Y. M., Carballo, D., Koskinas, K. C., Bäck, M., Benetos, A., Biffi, A., Boavida, J.-M., Capodanno, D., Cosyns, B., Crawford, C., Davos, C. H., Desormais, I., Di Angelantonio, E., Franco, O. H., Halvorsen, S., Hobbs, F. D. R., Hollander, M., … ESC Scientific Document Group. (2021). 2021 ESC Guidelines on cardiovascular disease prevention in clinical practice. *European Heart Journal*, *42*(34), 3227–3337. https://doi.org/10.1093/eurheartj/ehab484

**Supplementary Tables**

**Supplementary Table 1**. Individual characteristics comparison between included and excluded participants

|  | **Included** | **Excluded** | **p-value** |
| --- | --- | --- | --- |
| **N** | 1867 | 3014 |  |
| **Women** *(%)* | 957 (51.3) | 1732 (57.5) | <0.001 |
| **Age** (*years)* | 61.2 [15.8] | 64 [18] | <0.001 |
| **Current worker** *(%)* | 1135 (60.8) | 1305 (47.9) | <0.001 |
| **Monthly household income** *(%)* |  |  |  |
| Low (up to 4'999 CHF) | 434 (23.3) | 589 (31.8) | <0.001 |
| Moderate (5’000 to 9'499 CHF) | 813 (43.6) | 807 (43.6) |  |
| High (more than 9'500 CHF) | 620 (33.2) | 454 (24.5) |  |
| **Current smoker** *(%)* | 324 (17.4) | 531 (20.2) | 0.02 |
| **BMI** *(kg/m^2^)* | 26.2 [5.6] | 26.5 [6.4] | 0.02 |
| **Current alcohol consumer** *(%)* | 529 (28.3) | 593 (25) | 0.01 |
| **Arterial Hypertension** *(%)* | 423 (22.7) | 721 (23.8) | 0.35 |
| **Dyslipidemia** *(%)* | 1170 (62.7) | 1857 (61.4) | 0.36 |
| **Diabetes mellitus** *(%)* | 165 (8.8) | 367 (13.6) | <0.001 |
| **GFR (ml/min/m^2^)** | 81.1±14.5 | 80.5±15.8 | 0.15 |
| GFR > 90 (%) | 561 (30.1) | 374 (33.5) | 0.038 |
| GFR 90-60 (%) | 1160 (62.1) | 655 (58.7) |  |
| GFR < 60 (%) | 146 (7.8) | 86 (7.7) |  |
| **Cardiovascular treatments** *(%)* |  |  |  |
| Statin therapy | 335 (17.9) | 663 (21.9) | 0.001 |
| Diabetic therapy | 117 (6.3) | 280 (9.3) | <0.001 |
| Antihypertensive therapy | 470 (25.2) | 959 (31.7) | <0.001 |
| **Depression** *(%)* | 355 (19) | 470 (25.5) | <0.001 |
| **Benzodiazepine** *(%)* | 134 (7.2) | 297 (9.8) | 0.002 |
| **Previous ASCVD** *(%)* | 114 (6.1) | 273 (9) | <0.001 |
| <2 years before baseline | 25 (21.9) | 47 (17.2) | 0.28 |
| **Risk according to ESC-SCORE** *(%)* | 3.8±4.8 | 4.8±5.5 | <0.001 |
| Low-risk (<1%) | 637 (34.1) | 660 (26.09) | <0.001 |
| Intermediate-risk (1% - 5%) | 579 (31) | 671 (26.5) |  |
| High-risk (5% - 10%) | 256 (13.7) | 357 (14.1) |  |
| Very high-risk (≥10%) | 395 (21.2) | 842 (33.3) |  |

Results express the number of participants (%), mean ± SD or median [IQR]. Percentages are expressed by row. P-values were derived using Pearson chi-square or Student’s T test where appropriate. Exclusion criteria: 1897 participants of the CoLaus second follow-up did not participate in accelerometry. 18 participants were excluded for a non-wear of the accelerometer more than 20% of the time. 272 participants were excluded for wearing the accelerometer less than 7 days. 840 participants were excluded for missing co-variables.

ASCVD: atherosclerotic cardiovascular disease; overweight HDL: high-density lipoprotein; LDL: low-density lipoprotein; GFR: glomerular filtration rate; IQR, interquartile range; SD: standard deviation.

**Supplementary Table 2**. Characteristics of participants according to their cardiovascular risk level

|  | **Cardiovascular risk level according to ESC-SCORE1** | | | | | **Previous ASCVD** | | **No risk factors** | |
| --- | --- | --- | --- | --- | --- | --- | --- | --- | --- |
|  | **Low risk (<1%)** | **Intermediate-risk (1% - 5%)** | **High-risk (5% - 10%)** | **Very high-risk (≥10%)** | **P-value** |  | **P-value** |  | **P-value** |
| **N** (*%)* | 637 (34.1) | 579 (31) | 256 (13.7) | 395 (21.2) |  | 114 (6.1) |  | 455 (24.4) |  |
| **Women** *(%)* | 446 (70) | 252 (43.5) | 132 (51.6) | 127 (32.2) | <0.001 | 33 (29) | <0.001 | 304 (66.8) | <0.001 |
| **Age** (*years)* | 51.6 [7.2] | 61.4 [8.5] | 70.4 [6.3] | 72.3 [12.4] | <0.001 | 70.2 [11.9] | <0.001 | 52.5 [8] | <0.001 |
| **Current worker** *(%)* | 576 (90.4) | 386 (66.7) | 67 (26.2) | 106 (26.7) | <0.001 | 33 (29) | <0.001 | 394 (86.6) | <0.001 |
| **Monthly household income** *(%)* |  |  |  |  |  |  |  |  |  |
| Low (up to 4'999.-) | 91 (14.3) | 136 (23.5) | 73 (28.5) | 134 (33.9) | <0.001 | 34 (29.8) | 0.02 | 58 (12.8) | <0.001 |
| Moderate (5’000 to 9'499.-) | 262 (41.1) | 236 (40.8) | 121 (47.3) | 194 (49.1) |  | 55 (48.3) |  | 176 (38.7) |  |
| High (more than 9'500.-) | 284 (44.6) | 207 (35.8) | 62 (224.2) | 67 (17) |  | 25 (21.9) |  | 221 (48.6) |  |
| **Current smoker** *(%)* | 102 (16) | 115 (19.9) | 35 (13.7) | 72 (18.2) | 0.11 | 18 (15.8) | 0.65 | 0 (0) | <0.001 |
| **BMI** *(kg/m^2^)* | 24.6 [5.7] | 25.7 [4.9] | 25.7 [5.4] | 27.3 [6] | 0.006 | 26.5 [5.6] | 0.02 | 23.9 [5.6] | <0.001 |
| **Current alcohol consumer** *(%)* | 152 (23.9) | 176 (30.4) | 88 (34.4) | 113 (28.6) | 0.007 | 25 (21.9) | 0.12 | 116 (25.5) | 0.12 |
| **Arterial Hypertension** *(%)* | 53 (8.3) | 133 (23) | 71 (27.7) | 166 (42) | <0.001 | 40 (35.1) | 0.001 | 0 (0) | <0.001 |
| **Dyslipidemia** *(%)* | 31 (4.9) | 516 (89.1) | 227 (88.7) | 385 (97.5) | <0.001 | 112 (98.25) | <0.001 | 0 (0) | <0.001 |
| **Diabetes mellitus** *(%)* | 0 (0) | 0 (0) | 0 (0) | 165 (41.8) | <0.001 | 24 (21.1) | <0.001 | 0 (0) | <0.001 |
| **GFR** *(ml/min/m^2^)* | 87.5±11.5 | 83.7±11.1 | 72.0±13.5 | 73.1±17.2 | <0.001 | 71.8±11.8 | <0.001 | 85.8±11.9 | <0.001 |
| GFR > 90 (%) | 268 (42) | 205 (35.4) | 23 (9) | 65 (16.5) | <0.001 | 23 (20.2) | <0.001 | 172 (37.8) | <0.001 |
| GFR 90-60 (%) | 369 (57.9) | 374 (64.6) | 171 (66.8) | 246 (62.3) |  | 64 (56.1) | <0.001 | 277 (60.9) |  |
| GFR < 60 (%) | 0 (0) | 0 (0) | 62 (24.2) | 84 (21.3) |  | 27 (23.7) | <0.001 | 6 (1.3) |  |
| **Cardiovascular treatments** *(%)* |  |  |  |  |  |  |  |  |  |
| Statin therapy | 22 (3.5) | 66 (11.4) | 60 (23.4) | 187 (47.3) | <0.001 | 84 (73.7) | <0.001 | 0 (0) | <0.001 |
| Diabetic therapy | 0 (0) | 0 (0) | 0 (0) | 117 (29.6) | <0.001 | 17 (14.9) | <0.001 | 0 (0) | <0.001 |
| Antihypertensive therapy | 57 (8.9) | 105 (18.1) | 89 (34.8) | 219 (55.4) | <0.001 | 81 (71.1) | <0.001 | 37 (81) | <0.001 |
| **Depression** *(%)* | 136 (21.4) | 98 (16.9) | 46 (18) | 75 (19) | 0.253 | 17 (14.9) | 0.249 | 69 (15.2) | 0.016 |
| **Benzodiazepine** *(%)* | 28 (4.4) | 35 (6) | 26 (10.1) | 45 (11.4) | <0.001 | 12 (10.5) | 0.153 | 15 (3.3) | <0.001 |

Results express the number of participants (%), mean ± SD or median [IQR]. Percentages are expressed by row. P-values were derived using Pearson chi-square, Student’s T test or one-way ANOVA analysis where appropriate. No risk factors between hypertension, diabetes, BMI>30kg/m^2^, current smoking, dyslipidaemia (total cholesterol >7.5mmol/L or LDL >4.9mmol/L or triglyceridemia >5mmol/L or LDL cholesterol above the threshold of ESC-SCORE1 for lipid-lowering therapy [i.e. no lipid-lowering therapy, LDL >2.5 for intermediate and high-risk level, LDL>1.8 for very high-risk level]) and previous ASCVD.

ASCVD: atherosclerotic cardiovascular disease; ESC-SCORE1; Systematic COronary Risk Estimation from 2016 European Society of Cardiology guidelines; BMI, body mass index; LDL: low-density lipoprotein; GFR: glomerular filtration rate; IQR, interquartile range; SD: standard deviation.

**Supplementary Table 3.** Association between cardiovascular risk levels and PA adherence

|  | **Crude odds ratio (95% CI)** | **P-value** | **Adjusted odds ratio (95% CI)** | **P-value** |
| --- | --- | --- | --- | --- |
| **Previous ASCVD vs.**  **no previous ASCVD** | 0.5 (0.3-0.7) | <0.001 | 0.9 (0.6-1.4) | 0.6 |
| **ASCVD <2 years before baseline vs.**  **ASCVD>2 years before baseline** | 0.7 (0.3-1.9) | 0.5 | 0.2 (0.1-1.0) | 0.05 |
| **Very high-risk group vs.**  **low-risk group** | 0.2 (0.1-0.2) | <0.001 | 0.7 (0.4-1.2) | 0.2 |
| **No ASCVD risk factor vs.**  **presence of at least one ASCVD risk factors** | 3.1 (2.5-4.0) | <0.001 | 1.2 (0.6-5.6) | 0.5 |

Characteristics of participants, which were significantly associated with adherence in univariate analysis. were included in multivariable analysis for adjustment as shown in Figure 3.
